# Body Mass Index Impacts In Vitro Fertilization Stimulation

**DOI:** 10.5402/2011/929251

**Published:** 2010-10-27

**Authors:** Micah J. Hill, Steve Hong, John L. Frattarelli

**Affiliations:** ^1^Eunice Kennedy Shriver National Institute of Child Health and Human Development, 10 Center Drive, Bethesda, MD 20892, USA; ^2^Program in Reproductive and Adult Endocrinology, Eunice Kennedy Shriver National Institute of Child Health and Human Development, MD 20847, USA; ^3^Department of Obstetrics and Gynecology, General Leonard Wood Army Community Hospital, 126 Missouri Avenue, MO 65473, USA; ^4^Advanced Reproductive Medicine and Gynecology of Hawaii, 407 Uluniu Street, Suite 312, Kailua, HI 96734, USA

## Abstract

The objective of the study was to prospectively determine if body mass index (BMI) is predictive of live birth rates in patients undergoing IVF. The prospective study enrolled 117 infertility patients with the primary outcome measure being IVF success rates. Mean BMI did not differ between patients with successful outcomes and those without successful outcomes. There was a significant positive correlation between BMI and the number of stimulated follicles (*r* = 0.19, *P* < .05). A significant negative correlation between BMI and ampules of gonadotropins used (*r* = −0.25, *P* < .01) and between BMI and days of stimulation (*r* = −0.19, *P* < .05) was noted. These data demonstrate that women with an elevated BMI produce more follicles, stimulate quicker, and require less gonadotropins during IVF. However, BMI did not have a significant effect on pregnancy outcome rates.

## 1. Introduction

The published data regarding the effect of body mass index (BMI) on *In Vitro Fertilization *(IVF) cycles vary [1–13]. It has been proposed that extremes of BMI can affect IVF success rates [2, 13]. The extremes in body weight probably affect fecundity due to hormonal imbalances and ovulatory dysfunction [3, 4]. The association of BMI and baseline hormone levels has not been consistently demonstrated. Most studies evaluating BMI are retrospective with limited sample sizes and not designed to definitively demonstrate a correlation with BMI and IVF success rates.

BMI, defined as weight in kilograms divided by height in meters squared (kg/m^2^), is a measure commonly used to objectively assess obesity [14]. Excess weight (BMI ≥ 25 kg/m^2^) and obesity (BMI ≥ 30 kg/m^2^) are known contributors to menstrual irregularities, anovulation, and infertility; however, the effect of being overweight on the success of assisted reproductive treatment is less certain. The impact of BMI on controlled ovarian hyperstimulation parameters, such as amount of gonadotropins used, days of stimulation needed, peak estradiol levels, number or quality of oocytes, and pregnancy success is conflicting [1–13]. 

While many studies have demonstrated an association between IVF stimulation parameters and BMI, few have shown a significant impact on IVF pregnancy outcomes [2, 5, 7, 12]. We could find only one prospective study whose purpose was to evaluate the influence of BMI on IVF pregnancy outcome [13]. Salha et al. in a matched cohort design studied 100 patients and found that a BMI ≥ 26 kg/m^2^ was associated with a higher dose of gonadotropins, fewer oocytes, lower fertilization, and pregnancy rates [13]. Based on the published data, we designed a prospective study to examine whether BMI is an accurate predictor of IVF baseline values, stimulation parameters, and outcomes.

## 2. Materials and Methods

### 2.1. Population

Patients between the ages of 19 and 42 years were included independent of their diagnoses or prior reproductive history. Because of the poor response documented in the literature, patients with elevated FSH levels (≥12 mIU/ml) and women >42 years of age were excluded [15–17]. All women undergoing IVF at Tripler Army Medical Center who met inclusion criteria were counseled and invited to participate in the study. 

All patients received 35 *μ*g oral contraceptive pills for 21 to 35 days. During the last seven days of the oral contraceptives, the patients were started on a GnRH-a (Lupron, TAP Pharmaceuticals, North Chicago, IL) for a total of 14 days followed by stimulation with exogenous gonadotropins. When the largest cohort of follicles reached the 16 mm to 18 mm range, 10,000 units of human chorionic gonadotropin (hCG) were administered. Transvaginal follicular aspiration took place in 35 to 36 hours later. On postretrieval day 3 or 5, embryos were transferred vaginally with abdominal ultrasound guidance using a Wallace transfer catheter (Cooper Surgical, Shelton, CT). 

The study protocol was approved by the human use committee at Tripler Army Medical Center. Investigators adhered to policies for protection of human subjects as prescribed in 45 CFR 46.

### 2.2. Experimental Design

The main outcome measure was pregnancy outcome (pregnancy, cancellation, live birth, and pregnancy loss) during IVF. BMI was calculated on the patients' baseline evaluation day after pituitary suppression with oral contraceptives and prior to the start of gonadotropin stimulation. Transvaginal ultrasound to assess basal antral follicle number and ovarian volume was performed on day 3 of the patient's menstrual cycle the month prior to IVF. All ultrasounds were performed by the primary author (Frattarelli). 

BMI was compared with respect to patient age, diagnosis, number of follicles, number of oocytes retrieved, basal serum labs (FSH, LH, and estradiol), peak estradiol level (defined as the level of estradiol on the day of human chorionic gonadotropin administration), dehydroepiandrosterone sulfate (DHEAS), ampules of gonadotropins administered, days of stimulation, and outcome rates (pregnancy, cancellation, miscarriage, and live birth). All basal serum hormone measurements were made during a nonmedicated cycle within 3 months of initiation of the IVF cycle. 

Pregnancy was confirmed by a rising serum hCG at 4 weeks gestation followed by sonographic confirmation of cardiac activity at 6 weeks gestation. Patients were canceled for failing to produce ≥3 expanding follicles or failure to respond to gonadotropins with an adequate estradiol response of at least 300 pg/ml.

### 2.3. Laboratory Assays

The LH, FSH, Estradiol (*E*_2_), and Testosterone assays were all done with the Advia Centaur chemiluminescence system (Bayer Diagnostics, Tarrytown, New York). The inter- and intra-assay coefficients of variation were from 2.0 to 2.9% and from 0.3 to 2.7% for FSH, from 3.2 to 3.0% and from 1.5 to 2.9% for LH, from 5.3 to 11.3% and from 4.6 to 6.7% for *E*_2_, from 2.3 to 6.2% and from 1.4 to 4.7% for testosterone. The radioimmunoassay used for Androstenedione (Esoterix Inc., Austin, Texas) had inter- and intra-assay coefficients of variation from 3.0 to 4.6% and from 6.2 to 10.8%. The DHEAS assay was performed using an Immulite immunoassay (Diagnostic Product Corporation, Los Angeles, California). The inter- and intra-assay coefficients of variation for DHEAS were 20% for each.

### 2.4. Statistical Analysis

Based on our previous retrospective study, controlling for probability of a Type 1 error at alpha = 0.05 and a power of 0.80, a sample of 90 patients would be able to detect a 10% difference in mean BMI among patients who are either successful or unsuccessful in their pregnancy attempt during IVF [1]. To allow for cancellations and drop outs, 118 patients were scheduled to be consented for the protocol.

For normally distributed data, a *t*-test was used to compare the mean values between two groups. For data that were not normally distributed, a Mann-Whitney rank sum test was used to compare the mean values between two groups. A Kruskal-Wallis one-way analysis of variance on ranks was used to compare outcomes among multiple groups whose data were not normally distributed. Dunn's method was used for pairwise multiple comparison. Differences in outcome rates were analyzed using a Chi-square or two-tailed Fishers exact test where appropriate. Linear regression analysis assessed the correlation between linear data. 

Patients were grouped by BMI beginning at <20 kg/m^2^ and extending to ≥40 kg/m^2^ at increments of 1 kg/m^2^. The clinical outcome rates (implantation rate, pregnancy rate, miscarriage rate, and live birth rate) were then calculated for each increment. The clinical outcome rates were then evaluated with receiver-operator characteristic (ROC) curves to determine if there was an obvious break point or threshold at which there was a significant change in outcome rates. Contingency table analyses were used to evaluate the outcome rates above and below the selected threshold value. 

An alpha error of 0.05 was considered significant for all comparisons. Relative risk and 95% confidence intervals are displayed where appropriate. All data are reported as means with their associated standard deviations.

## 3. Results

One hundred seventeen women undergoing 117 IVF cycles met inclusion criteria and were counseled, consented, and included in the study. No patients were lost to followup or dropped out of the study. Patients enrolled had the following primary etiologies and associated BMI for their infertility: tubal factor (26%) (BMI = 26.8 ± 4.6 kg/m²), anovulation (20%) (BMI = 27.2 ± 6.3 kg/m^2^), unexplained infertility (18%) (BMI = 26.6 ± 6.0 kg/m^2^), male factor (17%) (BMI = 25.7 ± 3.9 kg/m^2^), endometriosis (9%) (BMI = 24.8 ± 5.1 kg/m^2^), diminished ovarian reserve (6%) (BMI = 23.9 ± 5.4 kg/m^2^), and uterine factor (4%) (BMI = 26.2 ± 4.9 kg/m^2^). BMI did not differ based on primary etiology (*P* = .45).

Table 1 summarizes the demographics and the prestimulation characteristics of the patient population. Table 2 summarizes the stimulation characteristics and IVF cycle outcomes of the patient population. The data were subdivided to evaluate the impact of overweight (BMI ≥ 25 kg/m^2^) and obesity (BMI ≥ 30 kg/m^2^) on prestimulation parameters, stimulation parameters, and IVF outcome. When evaluating the overweight population, a significant difference was noted in the number of antral follicles, peak estradiol levels, ampules of gonadotropins used for stimulation, number of days of stimulation, and number of follicles. No significant difference was noted for any parameter when evaluating the obese patient population.

The BMI of the study population ranged from 18.6 kg/m^2^ to 43.9 kg/m^2^ with a mean of 26.1 ± 4.8 kg/m^2^. Using linear regression analysis, BMI was compared to the demographics, pre- and poststimulation parameters. The results showed a significant positive relationship between BMI and the number of follicles noted by ultrasound prior to egg retrieval (*r* = 0.19, *P* < .05). A significant negative correlation between BMI and ampules used (*r* = −0.25, *P* < .01) and between BMI and days of stimulation (*r* = −0.19, *P* < .05) was noted. The correlation with other pre- and poststimulation parameters was not statistically significant.

**Figure 1:**
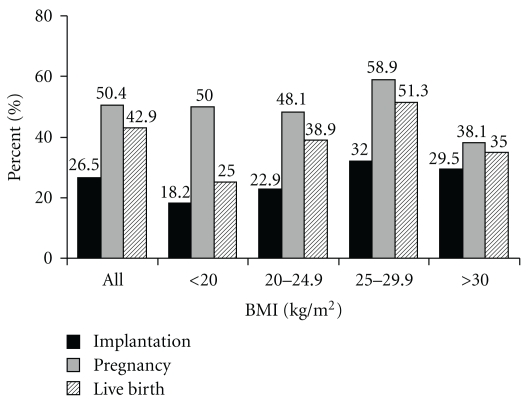
IVF outcome rates (Implantation, Pregnancy, Live Birth) for the entire patient population (ALL) and for the different subsets of the IVF population. The subsets are divided into thin (BMI < 20 kg/m^2^), normal weight (BMI = 20 to 24.9 kg/m^2^), overweight (BMI 25 to 29.9 kg/m^2^), and obese (BMI ≥ 30 kg/m^2^). Using contingency table analysis and receiver-operator characteristic curves, no significant threshold value was noted for outcome rates throughout the entire data range.

This study was powered to detect a 10% difference in BMI when evaluating pregnancy outcome. We examined the data by comparing the BMI of those who were able to achieve the outcome measure of pregnancy (BMI = 26.0 ± 4.6 kg/m^2^) compared to those not pregnant (BMI = 26.2 ± 5.1 kg/m^2^), no significant difference in BMI was noted. After grouping the patients by BMI beginning at <20 kg/m^2^ and extending to ≥40 kg/m^2^ at increments of 1 kg/m^2^, threshold analysis using contingency tables failed to identify any significant change in outcome rates at any of the BMI values. Outcome rates did not significantly differ according to BMI (Figure 1). ROC curves demonstrated an area under the curve of 0.53 (0.39, 0.63) for implantation rates, 0.50 (0.40, 0.61) for pregnancy rates, and 0.46 (0.29, 0.64) for miscarriage rates. 

Power analysis revealed that 4,169 patients would be needed to establish a statistical significance for BMI of < or ≥25 kg/m^2^ and pregnancy rates. While a change in pregnancy rates from 52.1% with a BMI < 30 kg/m^2^ to 38.1% with a BMI ≥ 30 kg/m^2^ is clinically significant, a power analysis revealed that 659 patients would be needed to establish a statistical significance.

## 4. Discussion

This study confirms the difficulty in establishing the effect of BMI on in vitro fertilization. With a prospective cohort design, we attempted to determine if mean BMI differed between patients with successful and unsuccessful IVF outcomes. We also evaluated the correlation of BMI to baseline and stimulation parameters during IVF.

Unlike some previous studies, which found it more difficult to stimulate follicular development in patients with an elevated BMI as demonstrated by an increase in number of days or ampules of gonadotropins required, our data demonstrated that fewer ampules of medication and days of stimulation were needed with increasing BMI [4–7]. These results suggest that as BMI increases, the amount of gonadotropins and days of stimulation decrease while the number of follicles produced increases. 

If our findings hold true, one might expect an increase in pregnancy rates. However, an increase in pregnancy rates was not realized in this study. In fact, the subanalysis of obese patients (BMI ≥ 25 kg/m^2^) suggested that clinically obese patients may experience a decrease in pregnancy rates. This finding is consistent with the results from previous studies [2, 5, 7, 13].

Salha et al. also prospectively evaluated BMI and patient outcome in IVF cycles and found that a BMI ≥ 26 kg/m^2^ was associated with a higher dose of gonadotropins, fewer oocytes, and lower fertilization and pregnancy rates [14]. Our data suggest that a lower dose of gonadotropins, more oocytes, and lower pregnancy rates are seen in patients with an elevated BMI. A possible explanation for the differences seen between the various studies could be the different subdivisions of BMI that have been used in studies to stratify patients. Previous studies have used up to five subdivisions of BMI; likewise, different definitions of obesity have been used (BMI > 25 kg/m^2^ versus BMI > 27 kg/m^2^) and a variety of different IVF induction protocols were used [2, 4, 5]. Our study looked at the entire range of BMI values in order to find a threshold value.

There are many strengths of this study. The most prominent is the use of prospective cohort design. Therefore, ascertainment and recall bias were minimized. No patients were lost to followup or dropped out of the study. All patients were included in the data analysis. All patients received the same GnRH-agonist protocol. Unlike the prior studies, we used threshold analysis to define a scientific cutoff point that would be predictive of success or failure. This use of threshold analysis decreases the bias that is found in arbitrarily choosing a BMI at which to study patients.

In summary, these data demonstrate that an increase in BMI may be associated with the need for fewer days of stimulation, fewer ampules of medication, and a greater number of follicles produced. Although BMI was associated with the named IVF stimulation parameters, BMI does not seem to significantly affect pregnancy outcome rates.

## Figures and Tables

**Table 1:** Demographics and Prestimulation IVF cycle Characteristics. Results are given as mean values with their associated standard deviations for all patients in the study, patients with BMI < 25 kg/m^2^ compared to patients with BMI ≥ 25 kg/m^2^, and patients with BMI < 30 kg/m^2^ compared to patients with BMI ≥ 30 kg/m^2^.

	All patients *N* = 117	BMI < 25 kg/m^2^ *N* = 58	BMI ≥ 25 kg/m^2^ *N* = 59	*P* value	BMI < 30 kg/m^2^ *N* = 96	BMI ≥ 30 kg/m^2^ *N* = 21	*P* value
Age	33.7 ± 4.1	34.1 ± 4.2	33.3 ± 4.0	.30	33.8 ± 4.1	33.1 ± 4.1	.44
D3 FSH (mIU/mL)	5.5 ± 1.7	5.7 ± 1.5	5.3 ± 1.9	.21	5.5 ± 1.7	5.5 ± 1.9	.95
D3 LH (mIU/mL)	5.3 ± 2.8	5.2 ± 2.4	5.4 ± 3.1	.90	5.3 ± 2.7	5.4 ± 3.1	.79
Androstenedione (ng/dL)	95.3 ± 50.1	88.2 ± 49.9	102.7 ± 50.4	.32	96.8 ± 48.0	86.4 ± 65.5	.61
DHEAS (ug/dL)	173.0 ± 96.9	166.9 ± 90.8	179.0 ± 103.0	.48	171.3 ± 89.4	181.2 ± 129.3	.86
Testosterone (ng/dL)	43.0 ± 18.9	40.3 ± 16.5	45.8 ± 20.7	.12	41.2 ± 16.5	51.6 ± 26.3	.14
Number of Antral follicles	20.6 ± 12.7	17.5 ± 10.0	24.2 ± 14.1	<.05*	19.7 ± 11.7	24.6 ± 14.7	.17
Total Ovarian volume (cm^3^)	13.4 ± 6.4	12.8 ± 6.4	13.9 ± 3.3	.48	13.1 ± 6.4	14.6 ± 6.7	.38

*Significant *P* values (≤.05).

**Table 2:** IVF Stimulation Characteristics and IVF outcomes. Results are given as mean values with their associated standard deviations for all patients in the study, patients with BMI < 25 kg/m^2^ compared to patients with BMI ≥ 25 kg/m^2^, and patients with BMI < 30 kg/m^2^ compared to patients with BMI ≥ 30 kg/m^2^.

	All patients *N* = 117	BMI < 25 kg/m^2^ *N* = 58	BMI ≥ 25 kg/m^2^ *N* = 59	*P* value	BMI < 30 kg/m^2^ *N* = 96	BMI ≥ 30 kg/m^2^ *N* = 21	*P* value
Peak estradiol (pg/mL)	2774.6 ± 1933.9	2476.7 ± 1866.7	3089.4 ± 1970.9	<.05*	2690.8 ± 1773.3	3171.6 ± 2586.6	.61
Ampules of gonadotropins	55.9 ± 11.8	59.3 ± 10.9	52.3 ± 11.8	<.001*	56.8 ± 11.7	51.9 ± 12.1	.13
Days of stimulation	10.1 ± 1.3	10.4 ± 12.2	9.7 ± 1.3	<.001*	10.1 ± 1.4	10.0 ± 1.0	.45
Number of follicles	18.9 ± 11.6	16.1 ± 10.3	21.9 ± 12.1	<.01*	17.8 ± 10.3	24.3 ± 15.5	.12
Number of oocytes	16.7 ± 11.9	15.5 ± 11.1	18.1 ± 12.6	.21	16.3 ± 11.5	19.0 ± 14.0	.56
Number of mature oocytes	13.2 ± 8.9	12.4 ± 8.9	14.1 ± 9.0	.24	13.1 ± 8.9	13.8 ± 9.3	.85
Number of embryos	10.4 ± 7.8	9.6 ± 7.4	11.3 ± 8.2	.18	10.4 ± 7.9	10.8 ± 7.8	.83
Number of embryos transferred	2.9 ± 0.9	3.0 ± 0.9	2.9 ± 1.0	.54	2.9 ± 0.9	2.9 ± 0.9	.80
Day of embryo transfer	3.6 ± 1.1	3.6 ± 1.1	3.6 ± 1.1	.93	3.7 ± 1.1	3.2 ± 1.0	.19
Pregnancy rate (%)	50.4%	48.3%	52.5%	.64	52.1%	38.1%	.30
Implantation rate (%)	26.5%	22.6%	30.7%	.10	25.7%	28.6%	.65

*Significant *P* values (≤.05).
